# Supplementary material for: MetaRibo-Seq measures translation in microbiomes
Source: Nat Commun. 2020 Jun 29;11:3268. doi: 10.1038/s41467-020-17081-z (PMC7324362; doi:10.1038/s41467-020-17081-z)
Supplement: Supplementary file 10 — Supplementary Data 7 [file 41467_2020_17081_MOESM10_ESM.zip › File2/Confidence_VeryHigh_Taxonomy/134806_out.krona.html]

Javascript must be enabled to view this page.

members
magnitude
magnitudeUnassigned
count
unassigned
taxon
rank

134806\_out

8

SRS014412\_contig\_number\_contig-100\_1274.109627SRS018984\_contig\_number\_9029SRS019693\_contig\_number\_7968SRS050752\_contig\_number\_13117SRS143070\_contig\_number\_2953SRS143895\_contig\_number\_41690SRS144506\_contig\_number\_55756SRS146813\_contig\_number\_contig-100\_181.147452
31

3

SRS014855\_contig\_number\_11578SRS053214\_contig\_number\_11967SRS146812\_contig\_number\_contig-100\_9618.228495

20
superkingdom
2

20
phylum
1239

2
species

SRS049712\_contig\_number\_19687SRS143466\_contig\_number\_32842
1947940

186801
class
18

order
18
186802

541000
6
family

genus
6
292632

1897022

SRS015663\_contig\_number\_27448SRS063127\_contig\_number\_271SRS1041092\_contig\_number\_5307SRS104311\_contig\_number\_31949SRS143085\_contig\_number\_contig-100\_137.49190SRS144362\_contig\_number\_43203
6
species

4
family
31979

1485
4
genus

1262824

SRS014459\_contig\_number\_38744SRS015065\_contig\_number\_contig-100\_2356.177358SRS103987\_contig\_number\_4053SRS104197\_contig\_number\_contig-100\_375.375
4
species


SRS017103\_contig\_number\_40627SRS055966\_contig\_number\_946SRS076804\_contig\_number\_14245SRS077730\_contig\_number\_17429SRS104084\_contig\_number\_3104SRS147346\_contig\_number\_61328SRS893279\_contig\_number\_15750SRS893373\_contig\_number\_27523
1897049
8
species
